# Missed positional gluteal compartment syndrome in an obese patient after foot surgery: a case report

**DOI:** 10.1186/s13037-020-00260-8

**Published:** 2020-09-15

**Authors:** Rami Khalifa, Madison R. Craft, Aaron J. Wey, Ahmed M. Thabet, Amr Abdelgawad

**Affiliations:** 1 Department of Orthopedic Surgery, Paul L. Foster School of Medicine, Texas Tech University Health Sciences Center, El Paso, TX 79905 USA; 2 Paul L. Foster School of Medicine, Texas Tech University Health Sciences Center, El Paso, TX 79905 USA; 3 Department of Orthopedic Surgery, Maimonides Medical Center, Maimonides Bone and Joint Center, 6010 Bay Parkway, Brooklyn, NY 11204 USA

**Keywords:** Gluteal compartment syndrome, Compartment syndrome, Buttock pain, Post-operative complication

## Abstract

**Background:**

Gluteal compartment syndrome is an uncommon condition and can be difficult to diagnose. It has been diagnosed after trauma, vascular injury, infection, surgical positioning, and prolonged immobilization from drug or alcohol intoxication. The diagnosis is based on clinical findings and, in most cases, recognizing these symptoms and making a diagnosis early is critical to a complete recovery.

**Case presentation:**

A 53-year-old male who underwent left foot surgery had severe pain to his contralateral hip and posterior gluteal compartment radiating to the right lower extremity immediately postoperative. He was positioned supine with a “bump” placed under his right hip to externally rotate his operative leg during the surgery. Due to the patient’s complex past medical history, a presumptive diagnosis of a herniated disc and/or compression of the sciatic nerve was made as a cause for the patient’s pain. This resulted in a misdiagnosis period of 36 h until the patient was diagnosed with unilateral gluteal compartment syndrome. Performing a fasciotomy was decided against due to the increased risk of complications. The patient was treated with administration of IV fluids and closely monitored. On post-op day 6, the patient was discharged. At three months post-op, the patient was walking without a limp and he had no changes in his peripheral neurologic examination compared to his preoperative baseline.

**Conclusion:**

Gluteal compartment syndrome is a surgical emergency that must be considered postoperatively especially in obese patients with prolonged operation times who experience acute buttock pain. The use of positional bars or “bumps” in the gluteal area should be used with caution and raise awareness of this complication after orthopedic surgeries.

## Background

Compartment syndrome in the gluteal area is a rare and often unrecognized syndrome. Trauma, vascular injury, infection, surgical positioning, and prolonged immobilization from drug or alcohol intoxication are the most common causes of gluteal compartment syndrome [1–4]. Early recognition and treatment can help to prevent long term complications such as residual sciatic nerve problems or renal failure from rhabdomyolysis [5, 6].

A systemic review of gluteal compartment syndrome found that 50% of the gluteal compartment syndrome cases were due to prolonged immobilization after alcohol or other drugs intoxication or after long surgical interventions such as total hip or knee arthroplasty or procedures with complications that prolong OR time [7]. The lithotomy and lateral decubitus positions are commonly used for orthopedic procedures and have been associated with gluteal compartment syndrome [8, 9]. Risk factors such as obesity in addition to extended operative time and patient positioning should warrant suspicion of gluteal compartment syndrome when symptoms arise. This article will describe a case of missed gluteal compartment syndrome that followed contralateral foot surgery. The report includes a literature review for positional gluteal compartment syndrome cases following orthopedic surgeries. We only indicated cases that occurred following orthopedic surgeries not performed in the pelvis/hip area so that the post-operative gluteal compartment syndrome is due to patient “positioning” and not due to “local trauma by the surgery”.

## Case presentation

### Pre-operative history

**Fig. 1:**
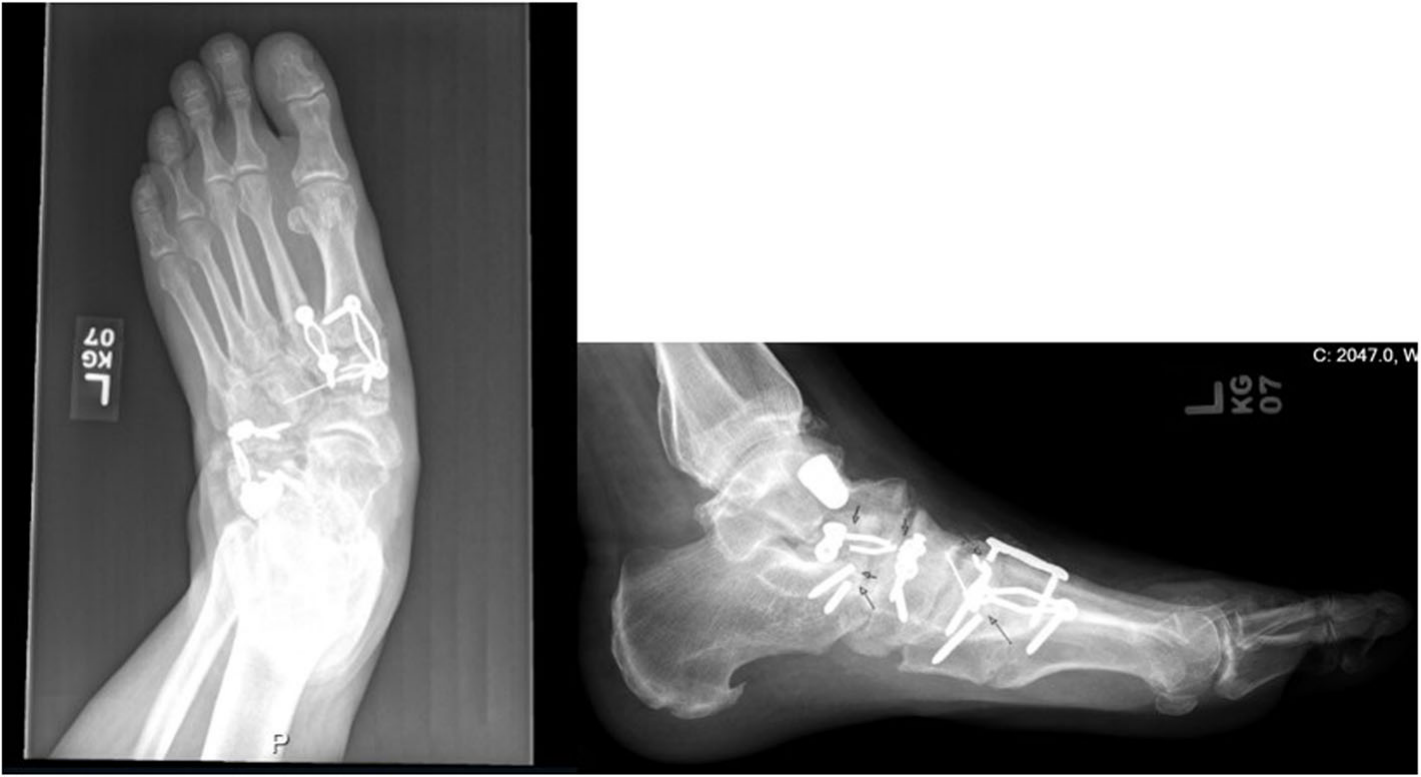
preoperative Radiographs showing failure of the fixation construct and the patient developed Charcot arthropathy of the midfoot

A 53-year-old male with diabetes mellitus and documented peripheral neuropathy presented with left foot pain and deformity. He had a previous Lisfranc injury treated with open reduction and internal fixation three years before presentation. The fixation construct failed and the patient developed Charcot arthropathy of the midfoot (Fig. 1). He also presented with stage III posterior tibial tendon insufficiency with a rigid hindfoot valgus deformity and forefoot abduction. The patient was indicated for hardware removal, medial column arthrodesis, and subtalar arthrodesis to correct the flatfoot deformity and a triple hemisection Achilles tenotomy for Equinus deformity. Past surgical history included lumbar spine decompression and fusion a few years prior. The patient had a weight of 149 kg and a height of 188 cm resulting in a BMI of 42.

### Operative technique

**Fig. 2:**
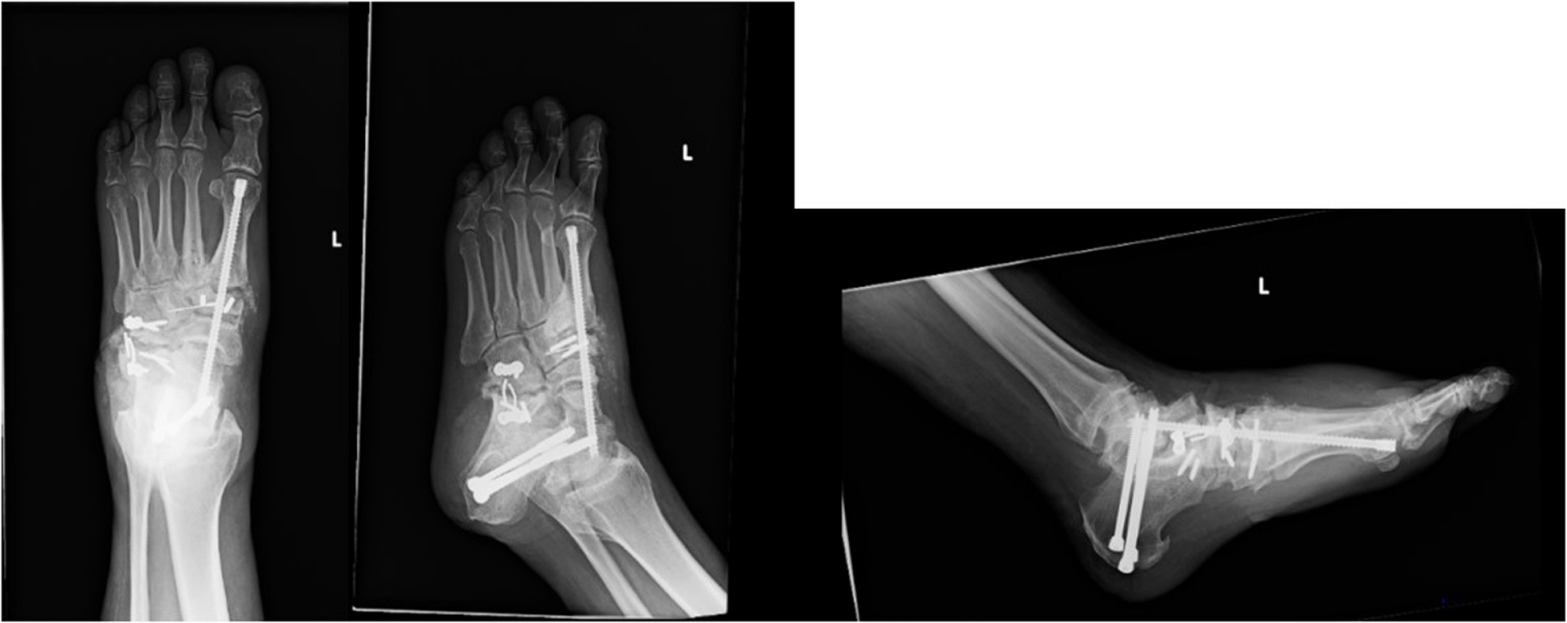
postoperative radiographs showing fusion of left foot medial column joints and the subtalar joint

In the OR, the patient was positioned supine on a regular operating table with a “bump” made of rolled up two sheets was placed under his right hip to externally rotate his operative leg. A left thigh tourniquet was placed and inflated to 350 mmHg for 120 min. No epidural or regional block was used. The duration of surgery was 3 h and 53 min. The procedure was performed in the following sequences 1) Complex hard ware removal (broken implant), 2) Precutaneous Achilles tendon lengthening, 3) Fusion of the 1st metatarsal with the medial cuneiform, 4) Fusion of the medial cuneiform with navicular bone, 5) Fusion of the navicular with the talus, 6) Fusion of the subtalar joint 96(Fig. 2). The procedure went uneventful. The patient was extubated and tolerated the procedure during surgery very well.

### Postoperative day (POD) 0

In the immediate postoperative period, the patient complained of 10/10 pain to the right (contralateral) hip and posterior gluteal compartment radiating to the right lower extremity. The patient was more comfortable with the bed at 45 ͦ compared to lying flat. An emergent CT scan was obtained to rule out a fracture of the femoral neck or proximal femur and it was negative and did not show any other abnormality around the pelvis area. Due to his prior history of lumbar spine surgery, degenerative changes in the lower lumbar spine with marked narrowing of disc space, and sclerosis of endplates and osteophytes were seen on CT, a presumptive diagnosis of a herniated disc and/or compression of the sciatic nerve was made. An urgent MRI was also ordered that night; however, the patient was unable to lay supine due to pain and the only way to obtain the MRI was under anesthesia. Due to his recent prolonged surgery, a decision was reached not to anesthetize the patient a second time for the MRI. The provisional diagnosis of radicular pain and sciatica was the prevailing diagnosis at that time. Please note that the diagnosis of the gluteal compartment syndrome was missed on the day of surgery. The patient experienced uncontrolled pain the night after surgery that did not respond to narcotic pain medication. He stated that the pain in his right lower back and buttocks was more severe than the pain in his left ankle from recent surgery.

### POD 1

**Fig. 3:**
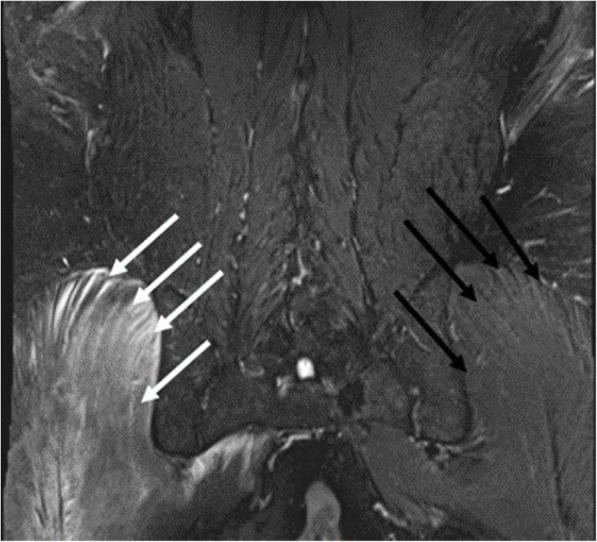
Patient’s MRI postoperatively of the lumbosacral region demonstrating hyperintense signaling from the right gluteal muscles (white arrows), please notice the difference between it and the normal left gluteal muscles (black arrows)

On POD 1, the patient continued to complain of radicular pain in the right L5, S1, and sciatic nerve distribution. On physical exam, he had grade 5/5 motor power strength with ankle dorsiflexion, plantar flexion, Extensor Hallucis longus, Flexor Hallucis Longus, knee extension, and knee flexion. The sensation was normal in the L1 to S1 nerve distribution. Dorsalis pedis and posterior tibialis pulses were palpable and symmetric in the left foot. The palpation of the right gluteal compartment was tense. The patient was also much more comfortable laying at 45° than supine in the bed. He was sent for an MRI of the lumbar spine and right hip. The MRI did not show a lumbar disc herniation but did reveal increased signal in the gluteal compartment (gluteus maximus, medius, and minimus) on the right side (Fig. 3).

### POD 2–3

Systemic blood work obtained on POD 2 included an elevated creatine kinase at 49,688 and a BUN/Cr at 44/1.5. The patient was treated with IV fluids and diagnosed with right gluteal compartment syndrome. Due to the prolonged misdiagnosis in the first 36 h post-op, the decision was made not to decompress the gluteal compartment to avoid operating on possibly necrosed muscle which would increase the risk for massive infection or significant fibrosis.

### POD 4–6

The patient was observed and treated without surgical decompression. His pain decreased gradually on each post-op day moving forward. Creatine kinase trended down to 12,064 on POD 5, and to 6702 on POD 6. His BUN/Cr improved to 25/1.0 on POD 6. After walking with physical therapy, the patient was discharged home on POD 6. At three months post-op, the patient was walking without a limp, had no proximal weakness, and had no changes in his peripheral neurologic examination compared to his preoperative baseline.

### Discharge protocol

Non-weight-bearing to left lower extremity and weight-bearing as tolerated to the right lower extremity. Physical therapy was scheduled to improve muscle recovery and regain strength. The patient was given forms with information on diabetic foot care, pain medicine instructions, walker use, fall prevention, and home safety. Outpatient clinic visits were scheduled every two weeks to monitor the patient’s recovery.

## Discussion

Gluteal compartment syndrome is a rare condition that mainly occurs after trauma directly to the gluteal area or prolonged immobilization with pressure on the gluteal region after intoxication or prolonged surgery [2–4, 10, 11]. Acute pelvic trauma can cause gluteal compartment syndrome due to local swelling from the injury that compromises vascular structures within a closed fascial space [1, 11, 12]. Postoperative gluteal compartment syndrome can occur due to local surgical trauma where structures have been incised and manipulated leading to edema; however, most cases of postoperative gluteal compartment syndrome affect the non-operative anatomical site due to prolonged immobilization and pressure.

A detailed literature review was performed to identify cases of positional gluteal compartment after orthopedic procedures. Cases due to direct trauma, vascular causes (e.g. injury to the gluteal vessels), intoxication, or those following non-orthopedic surgeries were excluded. We considered spinal procedures and extremity microsurgery among orthopedic surgeries. Cases of gluteal compartment syndrome following operative intervention on the ipsilateral hip (e.g. total hip arthroplasty) were considered “local trauma” and were excluded from this report. One article described bilateral gluteal compartment syndrome after total hip arthroplasty [13], we only included the contralateral side in the review as the ipsilateral side was considered local trauma. Thirteen case series/ case reports were included. Table 1 shows the details of the included papers/ cases. All studies were retrospective case reports [6, 10–17]. The average age was 53.2 years old, with most cases (84.6%) being males and the average BMI was 33.7 (from the studies that recorded BMI). The average operation time was 3 h.

**Table 1:** The literature review summary

Article/ Authors	Patient Characteristics (BMI, morbidities, obesity, weight, height)	Surgery performed and duration	Type of anesthesia	Positioning during surgery	The ipsilateral or contralateral side	Dialysis	Signs and symptoms that lead to the diagnosis	Treatment	Time after surgery fasciotomy was performed	Long term effects (complete recovery, persistent pain)
**Sarwar U, 2017** **[**11**]: Postoperative gluteal compartment syndrome following microsurgical free-flap hand reconstruction**	51yo F PMH: diabetes, obesity, hypertension, GERD	Microsurgical free-flap for hand - 8 h	General anesthesia	Supine	Contralateral (left buttocks)	No	Unremitting pain with radiation down the posterior thigh and leg. Restless and writhing in the bed unable to get comfortable. Localized tenderness. Passive movement, extension, flexion, abduction, and adduction, worsened the pain. Active extension and abduction of the hip were weak. Clinical diagnosis	Fasciotomy. A lumbar epidural provided much relief of sciatica type pain. It was removed after 2 day	3 h post-op.	Sciatica-type pain persisted along the right leg/foot after surgery, but the patient made a complete recovery
**Krysa J, 2002** **[**14**]: Gluteal compartment syndrome following PCL repair**	31yo M	PCL repair - 1 h	n/a	Modified lithotomy	Contralateral	No	Increasing pain in his contralateral buttocks despite stronger analgesia. Exacerbated by passive muscle stretching. The gluteal region was swollen, tense, and tender. Neuro/vascular normal. Intracomparemtneal pressure 58 mmHg while diastolic pressure at 40	fasciotomy	24 h post-op	Uneventful recovery with no neurological sequelae
**Osteen, 2012** **[**15**]: Bilateral gluteal compartment syndrome following right total knee revision**	52yo M PMH: obesity, HTN, HLD	Revision TKA - 3 h 20 min	Combined spinal-epidural	Supine	Bilateral	No	Agitation, severe distress from bilateral buttock pain. Left-sided buttock numbness. R/L gluteal region tense, hard, and erythematous. CPK/LFTs elevated. No passive movement toleration. Clinical diagnosis	Fasciotomy. Discontinued simvastatin POD 4. Vancomycin + Zosyn POD2	POD 2	No signs of necrosis during fasciotomy; discharged 5 days after fasciotomy with no neuro deficits or residual pain in the gluteal region
**Pacheco; 2001** **[**12**], Gluteal compartment syndrome after TKA with epidural postoperative analgesia** **Case 1**	47yo M PMH: obesity, HTN BMI: 41.66	TKA - 2 h and 15 min	Epidural	Supine	Bilateral	No	Back pain and difficulty in finding a comfortable position. Pyrexia (38.4 °C). swollen, tender, and painful buttocks. Not relieved by non-opioid analgesia. Compartment pressure results were at the borderline upper limit, clinically diagnosed	Fasciotomies	44 h post-op	Good recovery Discharged 9 days after admission; one year later, complained of gluteal discomfort on sitting
**Case 2**	71yo M weight: 81 kg BMI: 26.44	TKA (right) - 2 h 25 min	Epidural	Supine	ipsilateral	No	Right foot drop was noticed about 4.5 h after the epidural anesthesia had been discontinued. Examination showed loss of sensation in the distribution of the right sciatic nerve with no active movement of the ankle (foot drop). Swelling and tenderness of the right buttock and skin in the right gluteal area were indurated and discolored. The serum levels of urea and creatinine were elevated. Clinical diagnosis	Fasciotomy, sciatic nerve neurolysis. The necrotic muscle was excised from beneath the fascia lata and from vastus lateralis and rectus femoris	47.5 h postop	No change in motor or sensory function distal to knee; nerve conduction studies showed abnormalities in the sciatic nerve. Inability to obtain sensory or motor responses in the common peroneal and posterior tibial nerves.
**Rudolph, 2011,** **[**16**], Bilateral gluteal compartment syndrome and severe rhabdomyolysis after lumbar spine surgery**	65yo M height 178 cm, weight 124 kg, BMI 39.1 PHM: Obesity, DM2, HTN	bilateral decompression L3-L5–4 h	endotracheal anesthesia	Knee-chest position cushioned bar supporting the buttocks and soft side supports against the femoral trochanters	Bilateral	Yes	Pain in the buttocks had increased. Oliguria with darkened urine. Severe stiffness, tenderness, and painful swelling of gluteal muscles on both sides. Sciatic nerve palsy was absent. Creatine phosphokinase (CPK) level at 91,000 IU/l (normal 30–240 IU/l). Clinical diagnosis.	Fasciotomy, Hemodialysis despite the aggressive fluid replacement	7 h post-op	The patient required five courses of hemodialysis. Continued to have pain and was discharged dependent on a wheelchair due to moderate gluteal pain and bilateral insufficiency of gluteal muscles
**Polacek, 2009** **[**6**]: Gluteal compartment syndrome after lumbar laminectomy**	65yo M BMI 38, PMH: obesity, CKD III, DM2, HTN	Lumbar laminectomy Elbow - 4 h	Inhalation anesthesia	The elbow-knee position with side and buttock support	Left gluteal compartment	Yes	Increasing pain in both gluteal regions, especially on the left side. The pain did not respond to analgesic treatment. Both gluteal regions were abnormally firm and painful. Both feet had decreased capillary filling and felt cold. Palpable pulsation was found in the arteria dorsalis pedis (ADP) and arteria tibialis posterior (ATP), verified by Doppler ultrasound. No abnormal neurological findings were noted. Clinical diagnosis	Fasciotomy; Necrotic parts of gluteus muscles were removed	27 h post-op	Discharged 12 days after the initial operation (doesn’t say how the patient was doing or if walking)
**Somayaji, 2005** **[**13**]: Bilateral gluteal compartment syndrome after THA under epidural anesthesia**	39 yo M Congenital Hip Dysplasia	THA - 2 h	Epidural & general anesthesia	Left lateral position	Bilateral	No	Severe pain and discomfort in both buttocks 8/10. Continued to worsen and developed blisters in both buttocks during the night (second-night postop). Bilateral sciatic nerve palsy. Pedal pulses in both limbs. Sensation and power in both lower limbs failed to return to normal 24 h after cessation of epidural analgesia. Elevated creatinine kinase (31,000). Clinical diagnosis.	Fasciotomy with debridement of necrotic tissue	50 h post-op	Full-thickness skin grafts applied bilaterally, walks with a walking stick with minimal abduction and weak external rotation (the article doesn’t state if weakness in both sides or one side)
**Kumar V, 2007** **[**10**]: Gluteal compartment syndrome following joint arthroplasty under epidural anesthesia: 4 cases** **Case 1**	46yo F 101 kg, BMI: 38 PMH: obesity	L total knee arthroplasty - 2 h	Epidural	Supine	ipsilateral	No	Pain in the left buttock. Tense, tender swelling but no abnormal neurological findings. Clinical diagnosis.	Fasciotomy	48 h post-op	Complete recovery
**Case 2**	71yo M weight: 94 kg; BMI: 28	Left THA - 2 h 20 min	epidural	Right lateral position	contralateral	No	Severe right buttock pain. Firm, tense, tender swelling with erythema overlying the right buttock. Clinical diagnosis.	Fasciotomy	44 h post-op	Complete recovery
**Case 3**	55yo M weight: 86 kg BMI: 30	Right hip resurfacing arthroplasty - 3 h	epidural	Left lateral position	contralateral	No	Left buttock pain. Erythematous, tense, and tender area over the left buttock. Pain on passive flexion at the hip, Clinical diagnosis.	Fasciotomy	28 h post-op	Complete recovery
**Case 4**	72yo M weight: 81 kg BMI: 26	Right TKA - 2 h 25 min	Epidural	Supine	ipsilateral	No	Right foot drop. Loss of sensation along with the distribution of the sciatic nerve and no active movements at the ankle. Swelling and tenderness over the right buttock and serum potassium, urea, and creatinine were raised. Clinical diagnosis.	Fasciotomy	47 h post-op	Weak hip abductors with positive Trendelenburg sign and residual limp when walking at 18 months post-op but complete recovery of the sciatic nerve
**Mohanty, 2019** **[**17**]: Gluteal compartment syndrome a rare complication of lithotomy position and continuous postoperative analgesia**	27yo M BMI 36 PMH: obesity	R PCL repair - 2 h 30 min	Combined spinal-epidural	Modified Lithotomy position	Ipsilateral	No	severe pain in the right buttock whilst there was a recovery of sensory block. On examination, we noticed a tense, tender swelling in the right buttock. Clinical diagnosis	fasciotomy	18 h post-op	Uneventful recovery with a prolonged hospital stay

Six patients were positioned supine during surgery; two of the cases used lithotomy position; three cases with lateral positioning during hip surgeries had gluteal compartment syndrome develop on the lower side that patient laid on. There were two cases after lumbar decompression surgeries. In these two cases, the patients were positioned in the knee-chest/elbow position with “buttock support”. In our case, the procedure was performed in the supine position with elevation of the right side of the body with a “bump” under the right gluteal area to keep the left lower limb externally rotated. The use of a “bump” as well as the use of buttocks supports should be considered as a possible cause of gluteal compartment syndrome due to constant increased localized pressure.

Previous studies have found that major risk factors for gluteal compartment syndrome include obesity, prolonged operative time, and epidural anesthesia [3, 11, 18]. Our patient had a morbidly obese BMI of 42. This study’s literature review had patients with an average BMI of 33.7 indicating moderate obesity. This seems to be a factor contributing to the development of gluteal compartment syndrome due to elevated pressure from the increased patient weights and from prolonged surgeries that are typically associated with more obese patients. These cases require a lower threshold for suspicion postoperatively.

Nine patients in our review had epidural anesthesia used for the operation and/or postoperatively. It has been mentioned in the literature that epidural analgesia may predispose susceptible patients to gluteal compartment syndrome [12, 17]. In our case, the patient underwent the surgery under general anesthesia that why he had a severe pain shortly postoperative, this may support the use of general anesthesia in certain surgical interventions, which have the high risk of the position related compartment syndrome.

The most common clinical signs for gluteal compartment syndrome included pain in the gluteal region (92.3%), non-compressible tense swelling of the buttocks area with possible erythema (84.6%), pain that did not improve with analgesics (53.8%), pain elicited with passive range of motion of the hip (38.5%), and sensory/motor nerve dysfunction with the sciatic nerve distribution (38.5%). Four patients experienced foot drop (30.8%). Out of the 13 cases, only one was diagnosed based on measuring compartmental pressures and the other 12 were based on clinical findings and patient symptoms. All the published cases were treated with a fasciotomy. In our case, the gluteal and back of the thigh pain was the first and the main symptom of our case which developed immediately postoperatively, in addition to tense gluteal region on the physical examination.

The symptoms and signs of gluteal compartment syndrome can be aided by labs and imaging to rule out other causes of the presenting symptoms. Creatine kinase and potassium levels are often elevated due to muscle ischemia [19]. Thus, given the large muscle mass involved, it is likely that a rise in CK may be one of the signs of compartment syndrome. In our patient, the creatine kinase level was not recorded until POD 2 and was markedly elevated (more than 49,000). This high value indicated that significant tissue damage had already occurred. Earlier measurements of CK could have supported the diagnosis of gluteal compartment syndrome. In the literature review, two cases out of the 13 (15.4%) required dialysis due to renal affection by the circulating myoglobin from the muscle injury.

Our literature review revealed that the more time that elapses until fasciotomy, the worse outcome for the patient. All the published cases were treated with a fasciotomy. The average time after surgery to fasciotomy was 33.5 h. The patients that did not achieve a complete recovery had fasciotomies performed a minimum of 44 h after surgery. Thus, the diagnosis of gluteal compartment syndrome must be made promptly to treat as soon as possible and to achieve a better likelihood of a complete recovery. In many cases, due to the rarity of the condition, the masking of the symptoms from epidural anesthesia or more relevant issues from the primary surgery, the diagnosis may be delayed.

In our case, the diagnosis of position gluteal compartment syndrome was missed for the first 36 h as the patient was assumed to have radicular symptoms due to possible lumbar spine etiology. Once the diagnosis was confirmed, due to existing literature expressing more complications with late fasciotomies and fear of operating on necrosed muscle which carries a high incidence of infection, it was decided that a fasciotomy would not be performed [20]. Fortunately, the patient completely recovered with no sequelae from the compartment syndrome. This article should not be interpreted, by any means, as a message that we encourage managing these cases nonoperatively. It is simply a description of the course of the disease in our patient. In our literature review, Eight of the patient (61.5%) achieved a complete recovery while five patients (38.5%) had persistent symptoms including gluteal discomfort with pressure (2/13, 15.4%), sciatic nerve sensory or motor dysfunction (1/13, 7.6%), and gluteal muscles weakness with Trendelenburg sign (3/13, 23%) (One patient had 2 long term sequalae).

## Conclusion

Gluteal compartment syndrome is a surgical emergency that must be considered postoperatively especially in obese patients with prolonged operation times who experience acute buttock pain. The use of positional bars or “bumps” in the gluteal area should be used with caution and raise awareness of this complication after orthopedic surgeries.

## Data Availability

All materials (consent, pictures, labs) are available for review if needed.

## Abbreviations

MRI: Magnetic Resonance Imaging.
BMI: Body Mass Index
POD: Post-Operative Day
CK: Creatine Kinase
BUN/Cr: Blood Urea Nitrogen/Creatinine
